# A zwitterionic gel electrolyte for efficient solid-state supercapacitors

**DOI:** 10.1038/ncomms11782

**Published:** 2016-05-26

**Authors:** Xu Peng, Huili Liu, Qin Yin, Junchi Wu, Pengzuo Chen, Guangzhao Zhang, Guangming Liu, Changzheng Wu, Yi Xie

**Affiliations:** 1 Hefei National Laboratory for Physical Sciences at the Microscale, iChEM (Collaborative Innovation Centre of Chemistry for Energy Materials), Hefei Science Center (CAS), and CAS Key Laboratory of Mechanical Behavior and Design of Materials, University of Science and Technology of China, Hefei, Anhui 230026, China

## Abstract

Gel electrolytes have attracted increasing attention for solid-state supercapacitors. An ideal gel electrolyte usually requires a combination of advantages of high ion migration rate, reasonable mechanical strength and robust water retention ability at the solid state for ensuring excellent work durability. Here we report a zwitterionic gel electrolyte that successfully brings the synergic advantages of robust water retention ability and ion migration channels, manifesting in superior electrochemical performance. When applying the zwitterionic gel electrolyte, our graphene-based solid-state supercapacitor reaches a volume capacitance of 300.8 F cm^−3^ at 0.8 A cm^−3^ with a rate capacity of only 14.9% capacitance loss as the current density increases from 0.8 to 20 A cm^−3^, representing the best value among the previously reported graphene-based solid-state supercapacitors, to the best of our knowledge. We anticipate that zwitterionic gel electrolyte may be developed as a gel electrolyte in solid-state supercapacitors.

## Abstract (web summary)

Gel electrolytes are promising for solid-state supercapacitors, due to their favourable properties such as high ion migration rate, mechanical strength, and water retention. Here, the authors report on a zwitterionic gel electrolyte giving robust performance in graphene-based solid-state supercapacitors.

## Graphical abstract

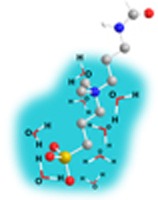

The development of highly efficient energy storage devices has greatly satisfied growing energy demands for our daily life, of which supercapacitors have emerged as high-performance energy storage devices for long operating lifetimes and high power densities^1^,^2^,^3^. Recently, gel electrolytes have attracted increasing attention in solid-state supercapacitors, due to their capability to fulfil multiple roles of electrolyte, separator and binder in solid-state supercapacitors^4^,^5^,^6^. Developing gel electrolytes accelerates the evolution of solid-state supercapacitors from traditional sandwich-type supercapacitors, to flexible, transparent and planar supercapacitors (micro-supercapacitors)^7^,^8^, and thus offering power support to flexible and even transparent electronics^9^,^10^. Generally, gel electrolytes are made of a polymeric material as matrix and an electrolyte salt to provide mobile ions^4^. Nowadays, non-aqueous gel electrolytes, such as block-copolymer-based gel electrolyte and silica-based gel electrolyte, have been developed dissolved in ionic liquid to solve the evaporation problem, achieving the enhancement in the ion migration rate and good mechanical strength, and making a great advance in electrochemical performance^11^,^12^,^13^,^14^. Aqueous gel electrolytes are dominantly based on polyvinyl alcohol (PVA) matrix, such as PVA/H_2_SO_4_ (refs 15, 16), PVA/KOH (refs 17, 18) and PVA/LiCl (refs 19, 20). The good abilities of PVA gel electrolyte, with a wide range of pH values like the aqueous electrolyte solution and serving as an elastic coating with a certain mechanical strength to avoid structure degradation of electrode materials, render excellent performance of PVA gel electrolytes for solid-state supercapacitors^21^. Despite the fact that PVA gel electrolytes offer convenience to fabricate solid-state supercapacitors, the development of aqueous polymeric gel electrolytes is still at a primary stage and the inner electrochemical mechanism remains to be explored. This leaves plenty of room to improve electrochemical performance of solid-state supercapacitors via chemical design of gel electrolytes.

Polyzwitterions are a type of charged polymer with robust water retention ability coming from the presence of a zwitterionic group in a repeat unit^22^, serving as a potential gel electrolyte catering for highly efficient solid-state supercapacitors. Polyzwitterions can be highly hydrated due to the strong interactions between the charged groups and water molecules, which make them suitable as a kind of superabsorbent polymeric material^23^. Also, their zwitterionic nature makes the cationic and anionic counterions of polyzwitterions easily separated during ion migration, ensuring a high ionic conductivity^24^,^25^. Moreover, polyzwitterions can form physical gels through the dipole–dipole interactions between the zwitterionic groups, thereby rendering the polyzwitterionic gel a certain mechanical strength^26^. In contrast to conventional polyelectrolytes, polyzwitterions usually exhibit the so-called ‘anti-polyelectrolyte' effect, thus it has a good solubility in aqueous solutions with a high salt concentration^27^,^28^. Besides, the charged and polar groups associated with polyzwitterions can strengthen the adhesion between the gel electrolyte and the electrodes, so that the polyzwitterionic gel electrolyte can be employed as a polymer binder to hold the electrodes together. More importantly, zwitterions have been used as electrolytes for high ionic conductivity and high lithium ion transfer number^29^,^30^,^31^,^32^. For example, zwitterionic gel electrolyte with 66.1 mS cm^−1^ with the help of high ion mobility of H^+^ was reported recently^33^. But there still presents great demand for introducing them to energy storage applications, especially for enhancing electrochemical performance via the electrolyte. In short, the combined advantages of water retention ability, high ion conductivity, reasonable mechanical strength and anti-polyelectrolyte effect renders polyzwitterions as a promising gel electrolyte for energy storage devices.

In this work, we develop a class of zwitterionic gel electrolyte, realising superior electrochemical performance in solid-state supercapacitors. In our case, the zwitterionic nature of poly(propylsulfonate dimethylammonium propylmethacrylamide) (PPDP) not only offers this gel electrolyte robust water retention ability at the solid-state through a combination of about eight water molecules around the charged groups but also brings ion migration channels to the electrolyte ions, leading to better electrochemical performance. When applying PPDP as a gel electrolyte, our graphene-based solid-state supercapacitor reaches a volume capacitance of 300.8 F cm^−3^ at 0.8 A cm^−3^, with a rate capacity of only 14.9% capacitance loss as current density increases from 0.8 to 20 A cm^−3^, representing the best value among the previously reported graphene-based solid-state supercapacitors, to the best of our knowledge.

## Results

### Chemical structures of PPDP/LiCl gel electrolyte

**Figure 1:**
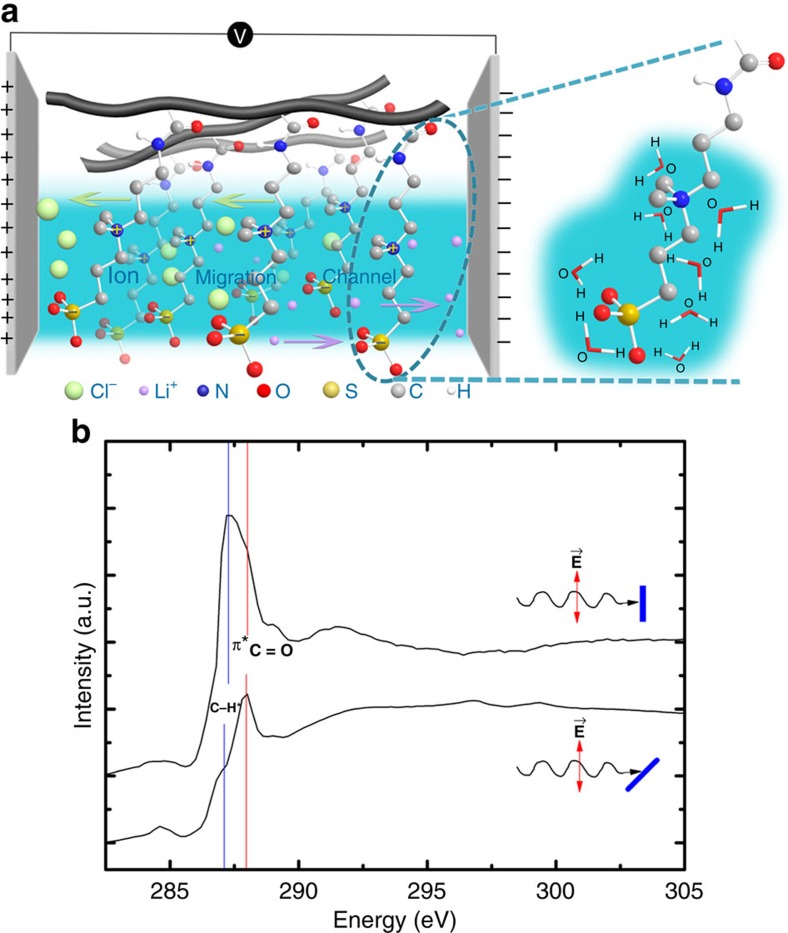
Zwitterionic PPDP under external electric field. (**a**) Schematic illustration of the PPDP gel electrolyte applied on electrodes. The ion migration channel is formed by applying external electric field. PPDP is strongly hydrated by water molecules with robust water retention ability due to the electrostatic interactions between the zwitterionic groups and water molecules. (**b**) Angular-dependent C K-edge X-ray absorption near-edge spectroscopy (XANES) of zwitterionic PPDP sample after applying external field, with linearly polarized soft X-ray beam.

PPDP is a kind of polyzwitterion bearing both positively charged quaternary ammonium group and negatively charged sulfonate group on the same monomeric unit. In this regard, PPDP is virtually neutral in a whole due to its zwitterionic character, as illustrated in Fig. 1a. Owing to the strong electrostatic interactions between the charged groups and water molecules, PPDP is highly hydrated by the surrounding water molecules with a robust water retention ability, showing a potential for superior electrochemical performance when supercapacitors operate at solid state. Meanwhile, the ion migration channel can be developed within the hydration layer along the polyzwitterion chains between two electrodes by applying external electric field due to the robust water retention ability of PPDP gel electrolyte. Such a kind of ion migration channel strongly boosts the efficiency of ion transport in the gel electrolyte. Therefore, the zwitterionic nature of PPDP makes the electrolyte ions of cationic Li^+^ and anionic Cl^−^ in gel electrolyte easily separated without overcoming the strong electrostatic attractions between the charged groups and the counterions during the ion migration and readily transferred onto the surface of graphene electrodes to realize the equivalent electric double-layer capacitance (EDLC), thereby enhancing the capacitance of solid-state supercapacitors.

Although the PPDP main chains are randomly dispersed in the gel electrolyte, the ion migration channel could be formed along the aligned zwitterionic side groups within the PPDP gel electrolyte induced by the external electric field. To verify the aligned zwitterionic groups in gel electrolyte under the external electric field, the angular-dependent X-ray absorption near-edge spectroscopy (XANES) is employed to probe the orientation of the molecular bonds, taking advantage of the polarized nature of the synchrotron radiation sources^34^. Figure 1b shows the total electron yield XANES spectra measured at the normal director (*θ*=90^o^) and *θ*=45^o^ while the PPDP sample was applied a potential. The spectra were normalized to the incident photon flux and further processed by levelling the pre- and post-edges^35^. The sample contains a lot of the carbon bonds, such as C–C, C–N, C=O, C–H and so on. So, the C K-edge XANES spectra present rich absorption features between 282 and 295 eV. Therein, the two main features of the XANES spectra at ∼287.3 and 287.9 eV present the significant angular dependence. For E-vector of the incident soft X-ray parallel to the surface of the substrate (*θ*=90^o^), the XANES presents a strong feature at 287.3 eV and a weak one at 287.9 eV. While in the case of oblique incidence of the X-ray beam (*θ*=45^o^), the former feature becomes weaker and the later becomes stronger. According to the reported literatures^36^, these two main features can be assigned to the excitations of the C–H antibond orbital (C–H*) and the C=O *π* antibond orbital (π*_C=O_). It is known that the C–H* molecular orbital is along the direction of the C–H axis and π*_C=O_ orbital lies two sides of the C=O axis. Therefore, the angular dependences of both XANES features indicate that the C–H and C=O bonds should be parallel to the substrate plane when a potential was applied. Since the main chains of the sample are fixedly attached on the surface of the substrate, these significant orientations of the C–H and C=O bonds point out that the zwitterionic side groups of the sample become ordered (vertical to the external field) in the electrochemical process. This fact indicates that the zwitterionic group can be aligned in some extent by the external electric field, accompanied by the formation of an ion migration channel in the gel electrolyte at solid state.

### Electrochemical performances of solid-state supercapacitor

**Figure 2:**
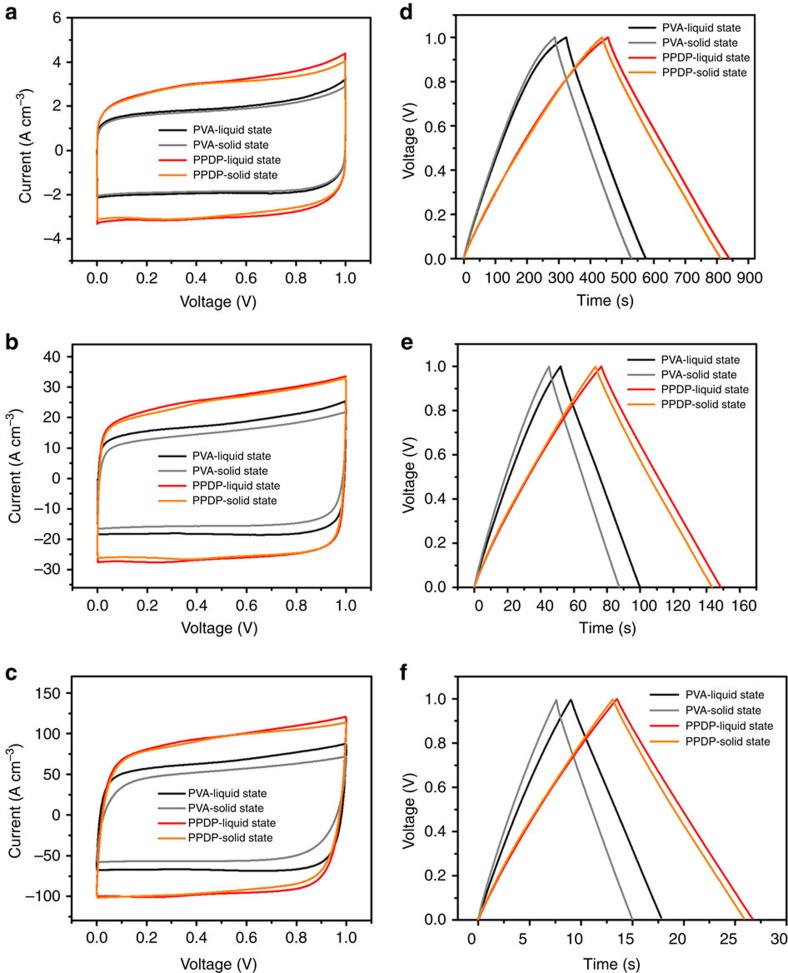
Electrochemical performance of graphene-based supercapacitors. (**a**–**c**) CV curves of graphene-based solid-state supercapacitors applying PPDP and PVA gel electrolytes at liquid state and solid state, the scan rates were 10, 100 and 400 mV s^−1^. (**d**–**f**) Galvanostatic charge–discharge curves of graphene-based solid-state supercapacitors applying PPDP and PVA electrolytes at liquid state and solid state at the current density of 0.8, 4 and 20 A cm^−3^.

**Figure 3:**
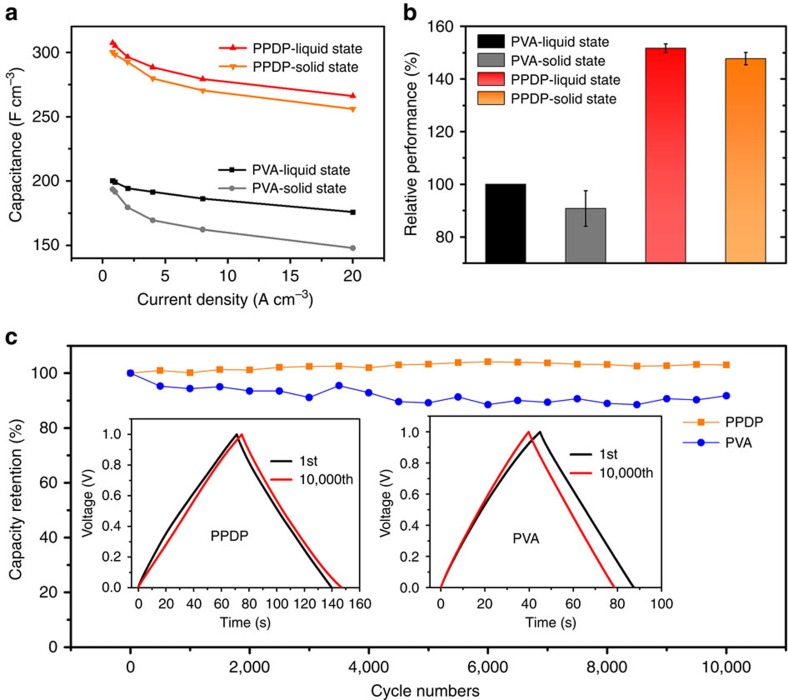
Capacitance and cycling performance of graphene-based supercapacitors. (**a**) Comparison of specific capacitance values for graphene-based supercapacitors between PPDP and PVA gel electrolytes at different current densities. (**b**) Relative performance of PVA and PPDP electrolytes in liquid and solid states, where the error bars are obtained based on the capacitances at different current densities. (**c**) Cycling performance of graphene electrodes applying PPDP or PVA gel electrolyte at solid state. Inset: comparison of CD curves between the 1st CD cycle and the 10,000th cycle for graphene-based solid-state supercapacitors applying PPDP (left of panel) and PVA (right of panel) gel electrolyte at 4 A cm^−3^.

The PPDP gel electrolyte with the ion migration channel and robust water retention ability significantly enhances the electrochemical performances of the as-fabricated graphene-based supercapacitor, as shown in Figs 2 and 3. To verify the superior electrochemical performances of the PPDP gel electrolyte, we conducted graphene-based symmetric supercapacitors and tested in a two-electrode configuration. Cyclic voltammetry (CV) was first studied with a potential range of 0–1.0 V at the scan rate of 10, 50, 100 and 400 mV s^−1^. As shown in Fig. 2a–c and Supplementary Fig. 1, the nearly rectangular CV curves indicated nearly ideal capacitive behaviours of graphene electrodes. Note that the resulting rectangle areas from the PPDP gel electrolyte are substantially larger than those for the PVA gel electrolyte, revealing that specific capacitance values of graphene-based supercapacitors applying PPDP gel electrolyte are much higher than those of PVA gel electrolyte. Considering that there is no pseudocapacitive material in electrode, the capacitance enhancement is mainly due to the better EDLC behaviour. Moreover, galvanostatic charge–discharge (CD) measurements were performed at the current density of 0.8, 1, 2, 4, 8 and 20 A cm^−3^ (Fig. 2e,f; Supplementary Fig. 2). The discharging times of PPDP sample were 384.0 s for liquid state and 376.0 s for solid state at the current density of 0.8 A cm^−3^, longer than those of PVA sample 250.4 s at liquid state and 242.0 s at solid state. The CD time of PPDP gel electrolyte sample continues to provide higher capacitance than that of PVA gel electrolyte sample. The as-fabricated graphene-based solid-state supercapacitor applying PPDP gel electrolyte reaches a volume capacitance of 300.8 F cm^−3^ at 0.8 A cm^−3^, recording the best value among the previously reported graphene-based solid-state supercapacitors to the best of our knowledge.

Accordingly, the as-fabricated supercapacitors applying PPDP gel electrolyte yielded specific capacitances of 300.8, 298.2, 292.4, 279.6, 270.4 and 256.0 F cm^−3^ at current densities of 0.8, 1, 2, 4, 8 and 20 A cm^−3^ at solid state, respectively, obviously larger than those of the as-fabricated supercapacitors applying PVA gel electrolyte, as summarized in Fig. 3a. Meanwhile, the as-fabricated supercapacitor applying PPDP gel electrolyte exhibited better rate capacity, with only 14.9% capacitance loss when the current density increases by a factor of 25 (from 0.8 to 20 A cm^−3^) compared with 23.6% loss for the as-fabricated supercapacitor applying PVA gel electrolyte. The PPDP gel electrolyte shows more advantages than the PVA gel electrolyte: (i) The capacitance loss of the PPDP gel electrolyte (1.4%) is much less than that of the PVA gel electrolyte (7.6%) after the electrolyte is transformed from liquid to solid state. (ii) The capacitance of as-fabricated supercapacitor applying PPDP gel electrolyte is 55.4% higher than the PVA gel electrolyte, as shown in Fig. 3b. The enhanced capacitance and rate capacity can be attributed to the unique property of PPDP gel electrolyte, which synergizes the effects of robust water retention ability and the ion migration channel with higher ion conductivity, and thus enhancing the capacitance, rate capacity and durability of the as-fabricated supercapacitor. In a word, applying the zwitterionic gel electrolyte, the graphene-based solid-state supercapacitor reaches a volume capacitance of 300.8 F cm^−3^ at 0.8 A cm^−3^ with a rate capacity of only 14.9% capacitance loss as the current density increases from 0.8 to 20 A cm^−3^. Cycling performance is a critically important characteristic for evaluating the stability of solid-state supercapacitors. Owing to the same electrode material in our as-fabricated symmetric supercapacitors, cycling performance is largely attributed to the long-term stability of gel electrolytes (Supplementary Figs 3 and 4; Supplementary Note 1). We tested the 10,000 times of charging and discharging cycles at 4 A cm^−3^. The PPDP gel electrolyte-based supercapacitor delivers a remarkable cycling stability of 103% retention up to 10,000 cycles, better than 91.8% retention of PVA gel electrolyte-based supercapacitor, as shown in Fig. 3c. These splendid electrochemical performances make such a kind of zwitterionic gel electrolyte attractive for fabricating the next generation of solid-state supercapacitors.

### Mechanism of PPDP gel electrolyte

**Figure 4:**
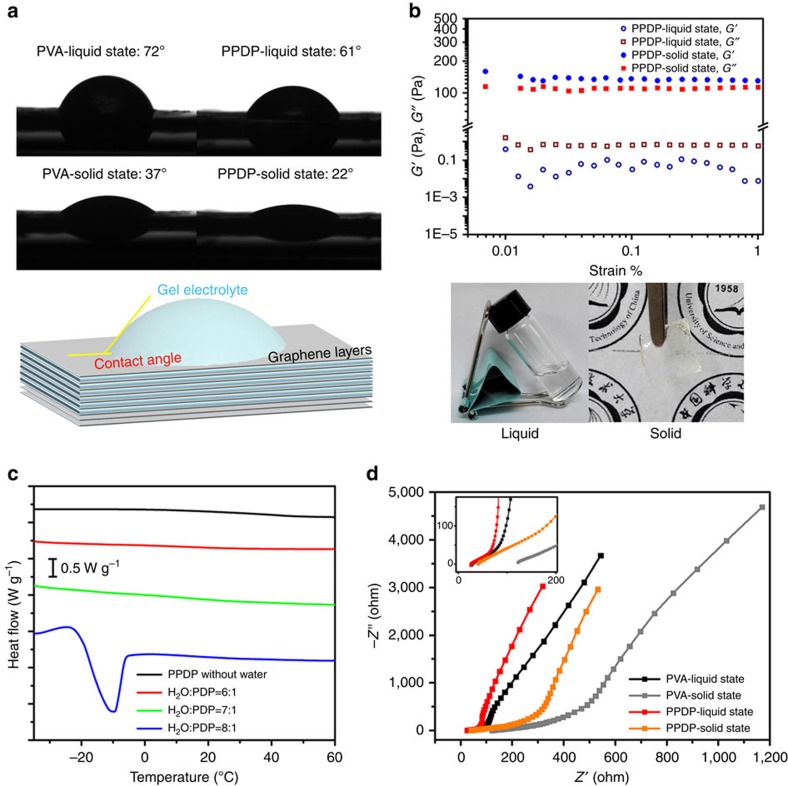
Physical and chemical mechanisms of PPDP gel electrolyte. (**a**) Static contact angles of PVA and PPDP gel electrolytes on the graphene electrode. Inset is the schematic illustration of the penetration of gel electrolyte into the multilayer graphene electrode. (**b**) Viscoelastic properties of the PPDP gel electrolyte at liquid and solid states. PPDP gel electrolyte is demonstrated at liquid state in a vial and at solid state with a freestanding solid thin film. (**c**) DSC thermograms of PPDP at different water contents. (**d**) Electrochemical impedance spectroscopy (EIS) of graphene-based solid-state supercapacitors applying PPDP and PVA gel electrolytes at liquid state and solid state.

To explore the physical mechanism of PPDP gel electrolyte, static contact angle and viscoelastic properties were tested to verify its favourable wetting behaviour and reasonable mechanical strength that is vital for construction of graphene-based solid-state supercapacitors. The affinity between the electrode and the gel electrolyte is crucial for supercapacitors because EDLC is strongly dependent on the surface contact areas between the electrode and the gel electrolyte. The wetting behaviour of the gel electrolytes on the graphene electrode is shown in Fig. 4a. For the initially prepared liquid-state electrolytes, the static contact angles are 72° and 61° for the PVA and PPDP electrolytes, respectively. The contact angles, respectively, decrease to 37° and 22° for the PVA and PPDP gel electrolytes after 24 h when the liquid state is transformed into the solid state due to evaporation. These facts suggest that the PPDP gel electrolyte can more effectively penetrate into the multilayer graphene electrodes and enhance the contact areas between the gel electrolyte and the working electrode compared with the PVA gel electrolyte as shown in the inset of Fig. 4a, due to the relatively low viscosity and high fluidity of the PPDP gel electrolyte than that of the PVA gel electrolyte (Supplementary Table 1). This characteristic of PPDP gel electrolyte would bring superior capacitance for solid-state supercapacitors. An ideal gel electrolyte not only can provide a superior electrochemical performance but also can serve as a binder with a certain mechanical strength to hold two electrodes together. Figure 4b shows the viscoelastic properties of the PPDP gel electrolyte. At the liquid state of PPDP electrolyte, the storage modulus (*G*′) is smaller than the loss modulus (*G*′′). In contrast, the *G*′ dominates over *G*′′ at the solid state, indicating that PPDP actually forms physically crosslinked gel due to the dipole–dipole interaction between the zwitterionic groups (Supplementary Fig. 5). The inter-chain hydrogen bonding between the amide parts of PPDP may also contribute to the formation of physical gel. Therefore, such a kind of gel with a storage modulus of 140 Pa provides enough mechanical strength to hold the two electrodes together, which can be supported from the formation of freestanding solid thin film by the PPDP gel electrolyte at a low water content as shown in the inset of Fig. 4b. Likewise, PVA gel electrolyte also exhibits similar viscoelastic properties at liquid and solid states (Supplementary Fig. 6; Supplementary Note 2).

To explore the chemical mechanism of PPDP gel electrolyte, differential scanning calorimetry (DSC) and electrochemical impedance spectroscopy were performed to verify the unique characteristics of robust water retention ability and efficient ion migration channel. The hydration capacity obtained from the DSC measurements demonstrates that PPDP has high water retention ability (Fig. 4c). Specifically, no endothermic peak can be observed in the thermogram during the heating of PPDP without water from −35 to 60 °C, suggesting that the polyzwitterion itself does not contribute to the thermal transition behaviour. Similarly, no thermal transition is observed during the heating process when the mole ratio of H_2_O to PDP equals 6:1 and 7:1, indicating that all the water molecules around one zwitterionic group (about eight water molecules) in the hydration shell are tightly bound to the polyzwitterion through the electrostatic interactions with the positively charged N^+^(CH_3_)_3_ and negatively charged SO_3_^−^ and no freezable water exists in the system. However, an obvious endothermic peak is observed as the mole ratio of H_2_O to PDP increases to 8:1, which means that the freezable water can be detected in the system when all binding sites of the polyzwitterion are saturated by water molecules^37^. Therefore, one PDP unit is strongly hydrated by seven to eight water molecules, as highlighted in Fig. 1. By contrast, Supplementary Fig. 7 and Supplementary Note 3 show that only one to two water molecules are tightly bound to one PVA monomeric unit via hydrogen bonding between the hydroxide group and the water molecules. Similar results are also observed in the low-field nuclear magnetic resonance (NMR) measurements (Supplementary Fig. 8; Supplementary Note 4). Consequently, the PPDP gel electrolyte gives rise to a higher water retention ability than PVA gel electrolyte at solid state (Supplementary Fig. 9; Supplementary Table 2; Supplementary Note 5). To confirm the efficient ion migration channel of the PPDP gel electrolyte, we performed electrochemical impedance spectroscopy tests using a two-electrode configuration from 100 mHz to 100 kHz. Usually, the intersection of the curve at the real part reflects the equivalent series resistance at the high frequency (100 kHz), which is contributed from the resistance of both electrolyte and electrode, while the linear region corresponds to the Warburg diffusion process (W), reflecting the ion diffusion into the electrode materials. As the Nyquist plots shown in Fig. 4d, the equivalent series resistance of PPDP-solid-state sample was 40.54 Ω, much smaller than that of PVA-solid-state sample of 122.6 Ω. Meanwhile, the slopes of PPDP gel electrolyte at liquid state and solid state are apparently larger than those of PVA samples at the low frequency, revealing that the transport and diffusion of ions into electrode materials of PPDP gel electrolyte are much better than those of PVA gel electrolyte, which answers for the superior electrochemical performance of the zwitterionic gel electrolyte.

**Figure 5:**
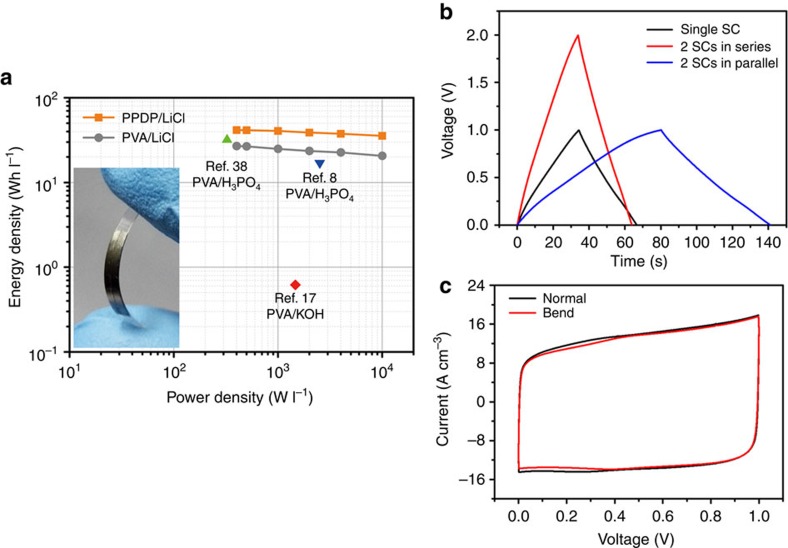
Applications of PPDP gel electrolyte. (**a**) A typical Ragone plot of the as-fabricated graphene-based solid-state supercapacitors. Inset is a prototype of graphene-based solid-state supercapacitor applying PPDP gel electrolyte with a planar configuration. (**b**) Galvanostatic charge–discharge curves of single supercapacitor (SC) and two SCs connected in series and in parallel at 8 A cm^−3^. (**c**) Comparison of CV curves between normal and bent status (90°), the scan rate is 50 mV s^−1^.

The ion migration channel along with the robust water retention ability of PPDP gel electrolyte greatly facilitates the ion transport during charging and discharging process. To further evaluate the energy efficiency of the as-fabricated graphene-based solid-state supercapacitors applying PPDP gel electrolyte, energy density and power density were calculated from CD curves. The Ragone plot of the as-fabricated graphene-based solid-state supercapacitors was shown in Fig. 5a. The as-fabricated solid-state supercapacitor applying PPDP gel electrolyte delivers a high energy density of 41.78 Wh l^−1^ at a power density of 400  W l^−1^, superior than 26.89 Wh l^−1^ of the as-fabricated solid-state supercapacitor applying PVA gel electrolyte, which is also excellent among other previously reported state-of-the-art graphene-based solid-state supercapacitors^8^,^17^,^38^, revealing that polyzwitterions especially PPDP would be a promising candidate to serve as gel electrolyte in solid-state supercapacitors. Besides, the feasibility of the as-fabricated solid-state supercapacitors is demonstrated in Fig. 5b. Generally, an effective way to enhance the potential or energy density can be achieved using serial and parallel assemblies. Compared with single supercapacitor, two as-fabricated supercapacitors in series show twice potential of 2.0 V, and two as-fabricated supercapacitors in parallel show almost twice CD time, indicating that the as-fabricated solid-state supercapacitor has good practical value. Meanwhile, there is an only slight difference in CV curves of the as-fabricated solid-state supercapacitor between normal and under bent status (90°), indicating that PPDP gel electrolyte has a certain mechanical strength with good flexibility and stability (Fig. 5c), which is suitable for establishing ultraflexible and even transparent energy storage devices and other electronics. The similar test about ionic conductivity of the PPDP gel electrolyte under different bending states is shown in Supplementary Fig. 10 and Supplementary Note 6. In addition, our proposed zwitterionic PPDP gel electrolyte also has general applicability for solid-state supercapacitors under acidic and basic conditions (Supplementary Fig. 11; Supplementary Note 7). Furthermore, the PPDP gel electrolyte has better self-discharging performance than that of the PVA gel electrolyte (Supplementary Fig. 12; Supplementary Note 8).

## Discussion

Zwitterionic gel electrolyte has been proven to be a promising gel electrolyte catering for solid-state supercapacitors. The zwitterionic nature of PPDP as gel electrolyte not only offers robust water retention ability but also brings ion migration channels to the electrolyte ions, leading to superior electrochemical performances. When applying PPDP as a gel electrolyte, the as-fabricated graphene-based solid-state supercapacitor reaches a volume capacitance of 300.8 F cm^−3^ at 0.8 A cm^−3^, with a rate capacity of only 14.9% capacitance loss when the current density increases from 0.8 to 20 A cm^−3^, recording the best value among the previously reported graphene-based solid-state supercapacitors, to the best of our knowledge. Moreover, the high water retention ability of the zwitterionic groups brings a robust cyclability, the capacitance remaining 103% retention after undergoing 10,000 CD cycles. We anticipate that zwitterionic gel electrolyte will be a promising gel electrolyte for the next generation of solid-state supercapacitors.

## Methods

### Preparation of gel electrolytes

The PVA/LiCl gel electrolyte was synthesized according to the procedure reported in previous literature^20^. An amount of 4.0 g PVA powder was put into 40 ml distilled water with stirring at 95 °C. After the PVA powder was completely dissolved, 4.0 g LiCl·H_2_O was added into the solution under vigorous stirring. When PVA/LiCl turned to be transparent and clear gel, it was cooled down to room temperature and the gel electrolyte was successfully prepared. The PPDP/LiCl gel electrolyte was prepared as follows. An amount of 1.0 g propylsulfonate dimethylammonium propylmethacrylamide (PDP), 1.0 mg 4,4′-azobis(4-cyanovaleric acid) (ACVA) and 4.0 ml distilled water were added into a round-bottom reactor. After three times of frozen–degassing–thawing cycles, the free-radical polymerization was conducted at 70 °C for 10 h. The polymerization is completed and cooled down to room temperature. Afterwards, the as-prepared PPDP was mixed homogeneously with 6.0 ml aqueous solution including 1.0 g LiCl·H_2_O under vigorous stirring to prepare the zwitterionic gel electrolyte, PPDP physical gel was successfully prepared.

To compare the electrochemical performances between PVA and PPDP gel electrolytes, the optimal initial inventory mass ratio of H_2_O:polymer:LiCl·H_2_O is fixed at 10:1:1 for the both two gel electrolytes as the concentration of LiCl·H_2_O has important influences on the physical state, ionic conductivity and specific capacitance of the gel electrolytes (Supplementary Figs 13 and 14; Supplementary Notes 9 and 10). And the link between ionic conductivity of gel electrolyte and the capacitance of solid-state supercapacitors is discussed in Supplementary Fig. 15 and Supplementary Note 11. The role of LiCl in enhancing the water retention of gel electrolytes is clarified in Supplementary Fig. 16 and Supplementary Note 12.

### Fabrication of graphene electrodes and solid-state supercapacitors

Graphene was obtained from the chemical reduction of graphene oxides. Graphene oxide was synthesized in a modified Hummer's method^39^. Then, graphene was reduced using hydrazine according to a reported method^40^. The graphene thin film on the surface of cellulose acetate membrane was obtained by a vacuum filtration process, followed by tightly pressing thin film onto polyethylene terephthalate (PET) substrate and slowly peeling off the cellulose acetate membrane, leading to freestanding graphene thin film on PET. Then, the graphene thin film was scraped along the channel to fabricate working electrodes, while two columns of Au current collectors were thermally evaporated on each side of the working electrodes. Finally, the solid-state supercapacitor was finally established after coating PPDP/LiCl gel electrolyte on the parallel region filling the channel between two working electrodes. The working electrodes were prepared with the same thickness and the surface areas of graphene as well as the gel electrolyte were applied onto the graphene with the same amounts for PVA and PPDP gel electrolytes, so that the electrochemical behaviour between PVA and PPDP gel electrolytes can be compared fairly. After the gel electrolyte became solid state, the graphene-based solid-state supercapacitor was fabricated. The volume of the graphene electrodes was calculated through multiplying the thickness by areas. The exact thickness of the graphene electrodes was ∼250 nm determined by the field-emission scanning electron microscopy on a FEI Sirion-200 SEM instrument (Supplementary Fig. 17). The distance between the two electrodes is ∼150 μm (Supplementary Fig. 18) and the exact surface area of each graphene electrode is 10 × 1 mm (Supplementary Fig. 19; Supplementary Note 13).

### Electrochemical tests

Electrochemical performances of the graphene-based supercapacitors were measured in a two-electrode system by CV and galvanostatic CD at an electrochemical station (CHI 760E). Potential range set for CV and CD tests was from 0 to 1.0 V. The as-fabricated graphene electrodes were directly used as the working electrodes for the electrochemical tests. Electrochemical impedance measurements of the supercapacitors were performed in the same configuration from 100 mHz to 100 kHz with a Zahner IM6 electrochemical workstation. The cycling stability of the as-fabricated supercapacitors was performed at room temperature with a sweep charge and discharge rate at the current density of 4 A cm^−3^ for 10,000 cycles.

### Other characterizations

Proton NMR (^1^H NMR) spectra were recorded on a Bruker AV400 spectrometer, using deuterium water as the solvent and tetramethylsilane as an internal standard. The ^1^H NMR spectra of PPDP and PVA were shown in Supplementary Fig. 20 and Supplementary Note 14. The number-average molar mass (*M*_n_=1.87 × 10^5^ g mol^−1^, *M*_w_/*M*_n_∼1.6) of PPDP was determined by size-exclusion chromatography (Waters 1,515), using monodisperse poly(ethylene glycol) as the standard and a 1.0 M NH_4_NO_3_ solution as the eluent with a flow rate of 1.0 ml min^−1^. The viscoelastic property of PPDP gel electrolyte was determined by a rheological measurement on a TA AR-G2 rheometer. The storage modulus (*G*′) and loss modulus (*G*′′) were measured as a function of strain with 1 Hz frequency at 25 °C. The DSC scans were conducted on a TA Instruments Q2000 scanning calorimeter at a heating rate of 5 °C min^−1^. The angular-dependent C K-edge XANES spectra, using the linearly polarized X-ray beam, were measured at the beamline BL12B of National Synchrotron Radiation Laboratory (NSRL, Hefei) in the total electron yield mode by collecting the sample drain current under a vacuum better than 10^−7^ Pa. The beam from the bending magnet was monochromatized utilizing a varied line-spacing plane grating and refocused by a toroidal mirror. The energy range is 100–1,000 eV with an energy resolution of ca. 0.2 eV. The PPDP sample was applied CV from 0 to 1.0 V for 1 h at the scan rate of 10 mV s^−1^ and underwent freeze-drying process before the measurement of XANES spectra. Low-field NMR measurements were performed by Bruker Minispec MQ-20 NMR analyser operating at a resonance frequency of 20 MHz (0.47 T). Sample was inserted in the NMR probe. Spin–Spin relaxation time, T2, was measured using the Carr-Purcell-Meiboom-Gill (CPMG) sequence.

### Data availability

The authors declare that the data supporting the findings of this study are available within the article and its Supplementary Information files.

## Additional information

**How to cite this article:** Peng, X. *et al*. A zwitterionic gel electrolyte for efficient solid-state supercapacitors. *Nat. Commun.* 7:11782 doi: 10.1038/ncomms11782 (2016).

## Supplementary Material

Supplementary Information Supplementary Figures 1-20, Supplementary Tables 1 & 2, Supplementary Notes 1-14 and Supplementary References.
