# Supplementary material for: A zwitterionic gel electrolyte for efficient solid-state supercapacitors
Source: Nat Commun. 2016 May 26;7:11782. doi: 10.1038/ncomms11782 (PMC4894970; doi:10.1038/ncomms11782)
Supplement: Supplementary Information — Supplementary Figures 1-20, Supplementary Tables 1 & 2, Supplementary Notes 1-14 and Supplementary References. [file ncomms11782-s1.pdf]

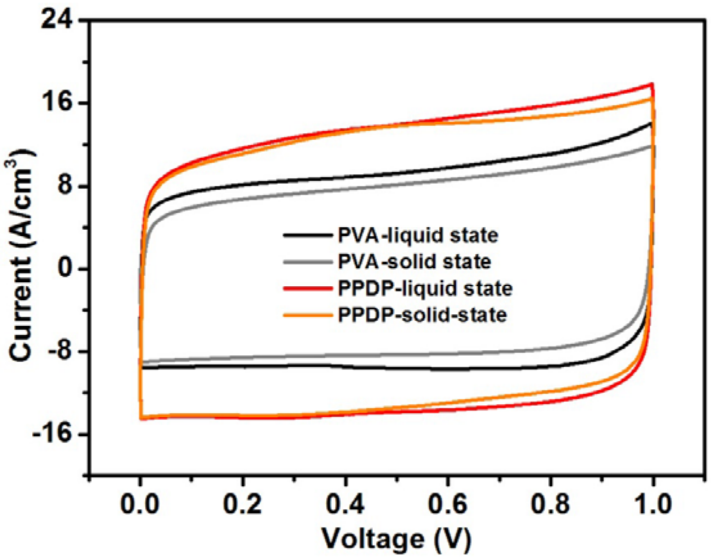

Supplementary Figure 1 | Cyclic voltammetry curves of the graphene-based solid-state supercapacitors applying PPDP and PVA electrolytes at liquid state and solid state with the scan rate of 50 mV s^{-1}.

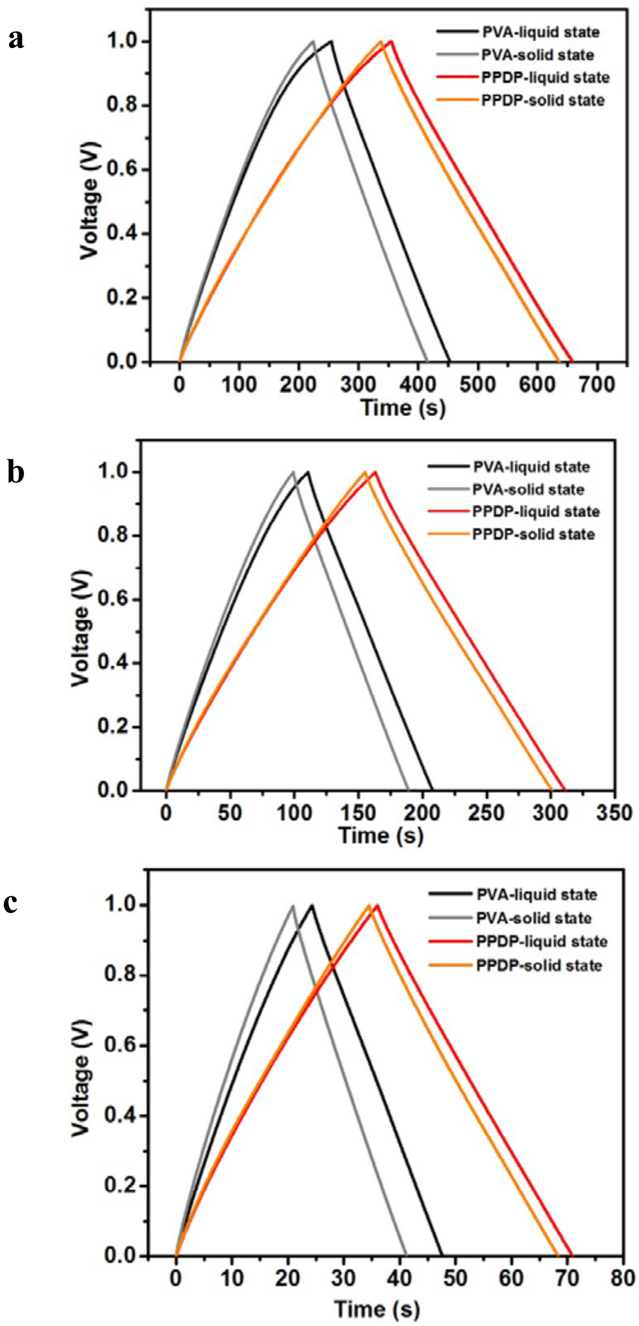

Supplementary Figure 2 | Galvanostatic charge-discharge curves of the graphene-based solid-state supercapacitors applying PPDP and PVA electrolytes at liquid state and solid state at the current density of (a) 1 A cm^{-3}, (b) 2 A cm^{-3} and (c) 8 A cm^{-3}.

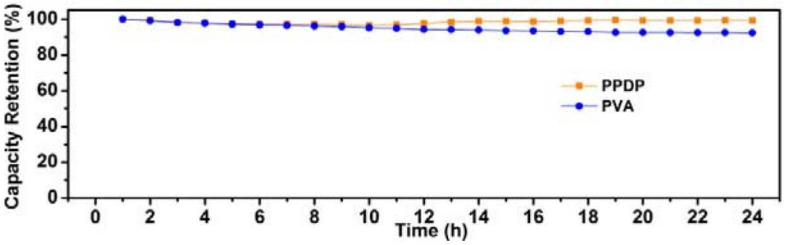

Supplementary Figure 3 | Capacitance retention of the as-fabricated graphene-based supercapacitors during the transformation of gel electrolyte from liquid state to solid state within 24 h. The capacitance is evaluated using galvanostatic charge/discharge curves at 4 A cm^{-3}.

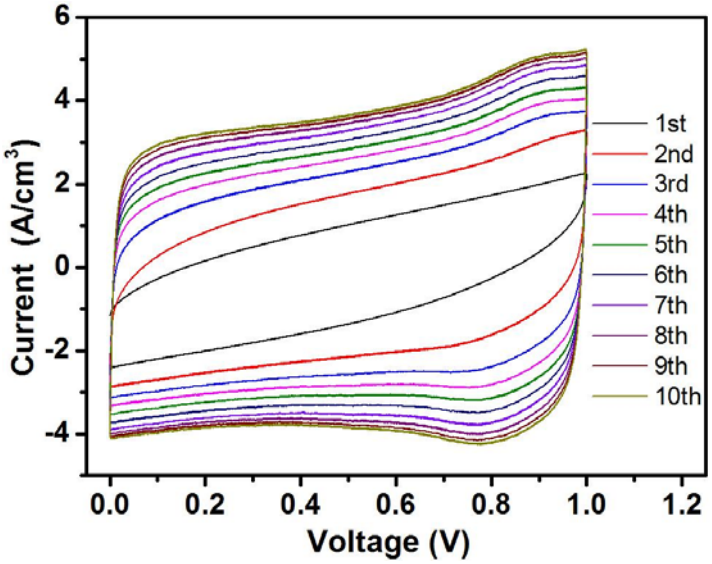

Supplementary Figure 4 | Cyclic Voltammetry of solid-state supercapacitor applying PPDP gel electrolyte from 1st to 10th cycles at the scan rate of 10 mV s^{-1}.

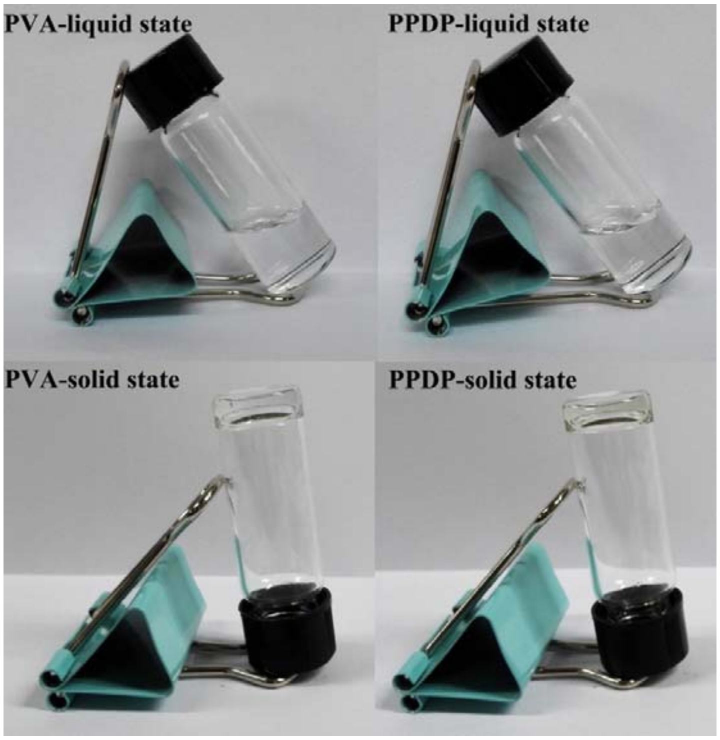

Supplementary Figure 5 | Demonstration of PVA and PPDP gel electrolytes.

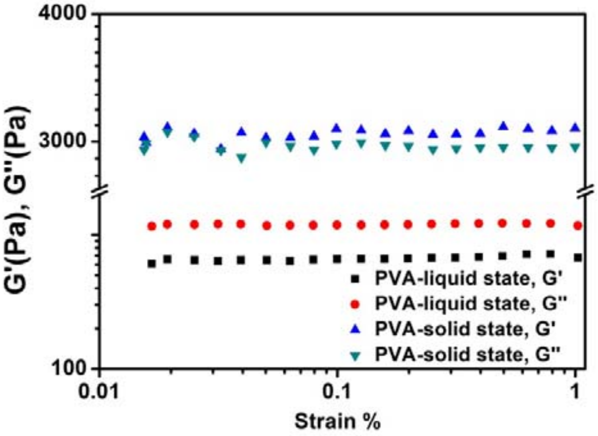

Supplementary Figure 6 | Viscoelastic properties of the PVA gel electrolyte at liquid and solid states.

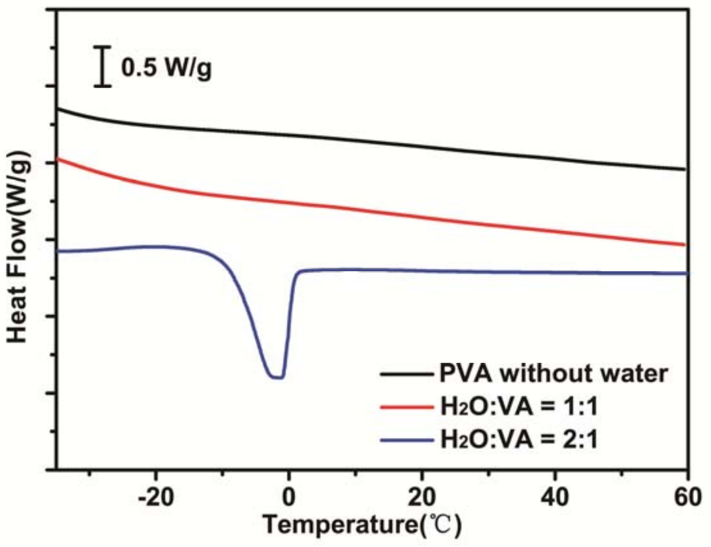

Supplementary Figure 7 | DSC thermograms of PVA at different water contents.

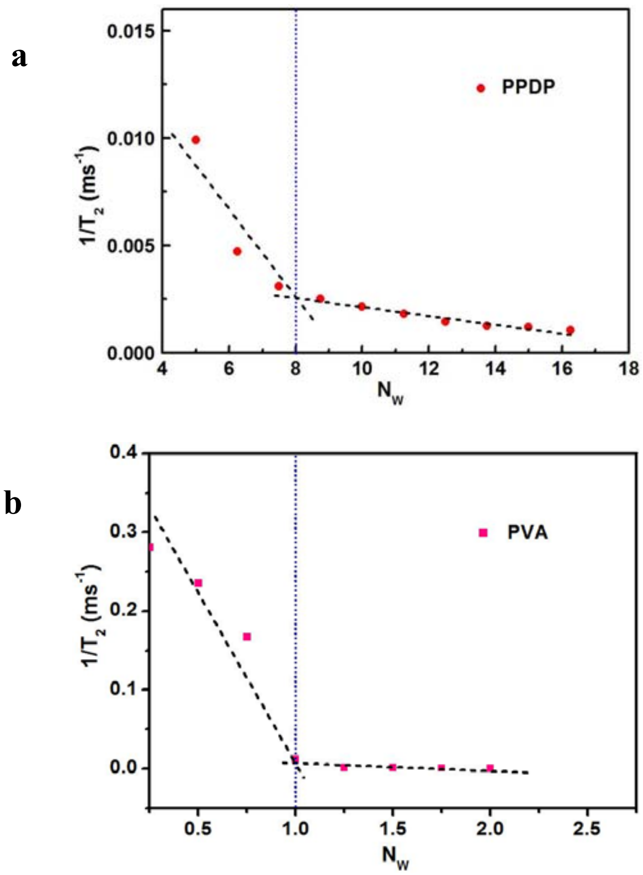

Supplementary Figure 8 | The reciprocal of spin-spin relaxation time ($1/T_2$) of the water component of PPDP (a) and PVA (b) as a function of the number of added water molecules per monomeric unit (N_w).

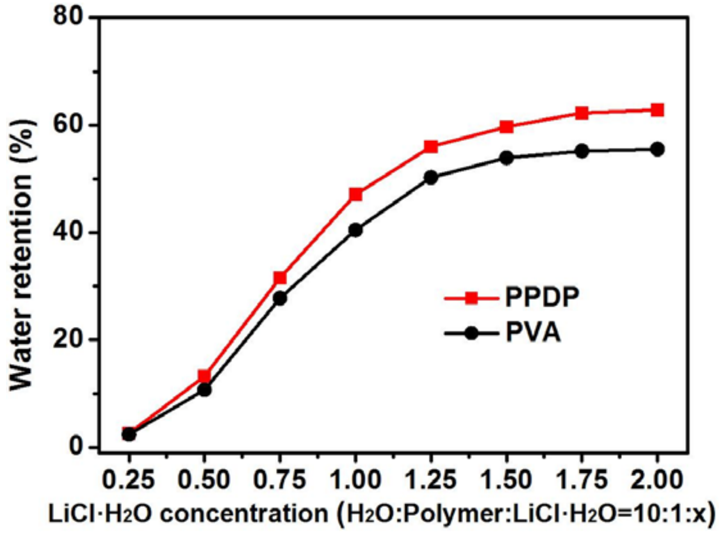

Supplementary Figure 9 | Water retention test of the PPDP gel electrolyte and PVA gel electrolyte with different salt concentration of LiCl•H_2O.

a

b

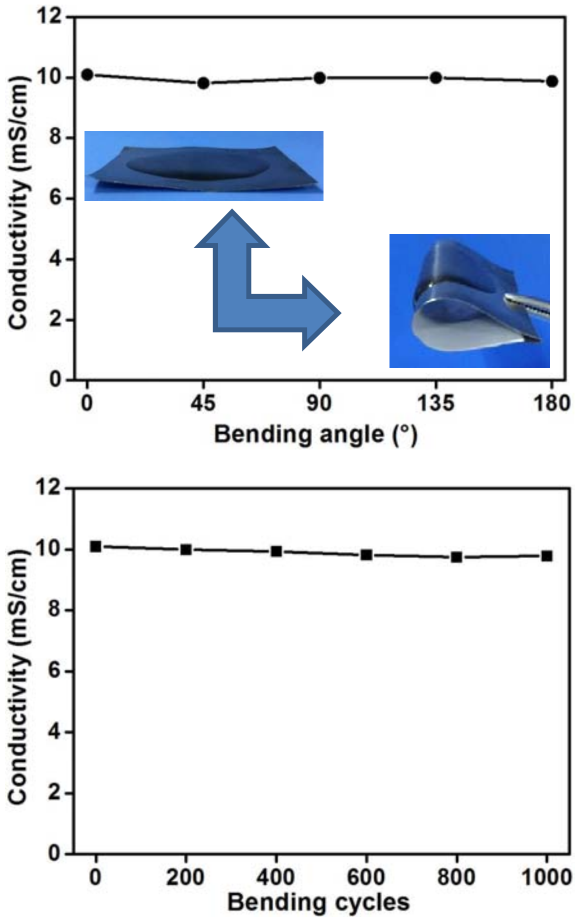

Supplementary Figure 10 | Bending test of PPDP gel electrolyte. (a) Conductivity of PPDP gel electrolyte at solid state under different bending states (45º, 90º, 135º, 180º). (b) Conductivity of PPDP gel electrolyte at solid state after different bending cycles.

a

b

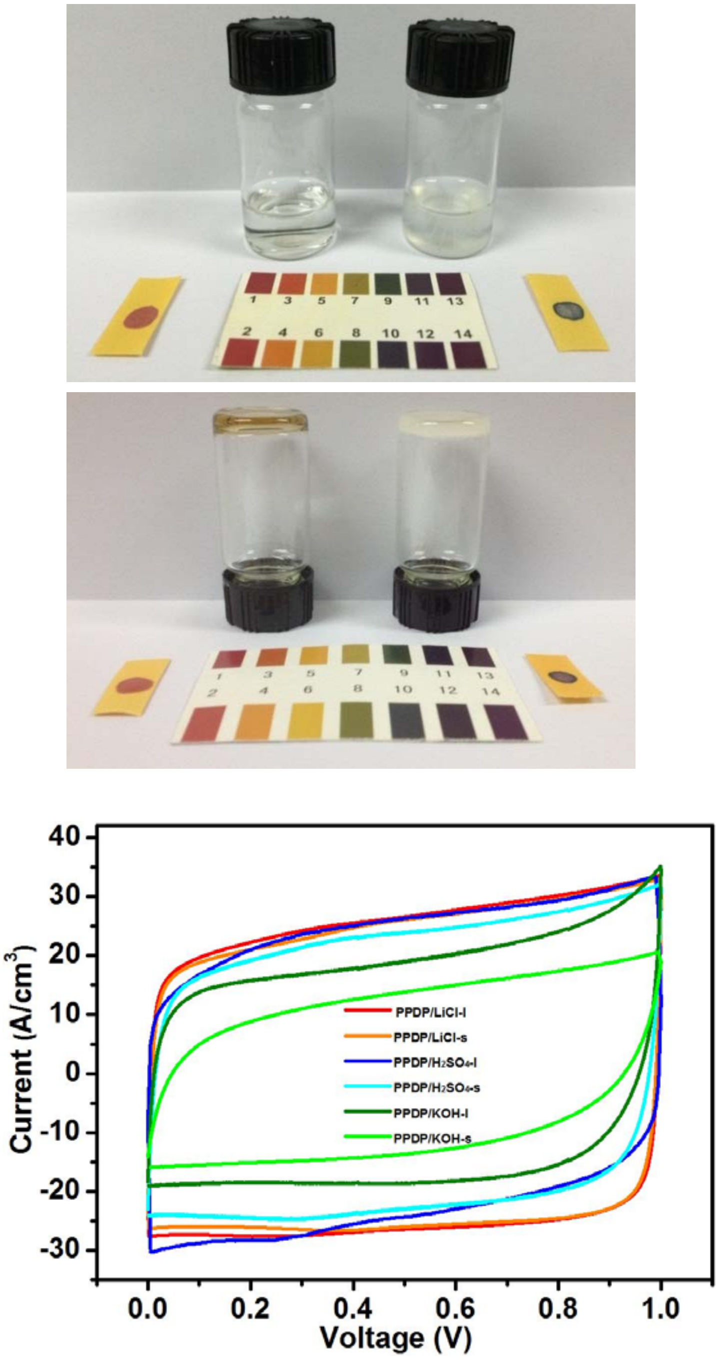

Supplementary Figure 11 | General applicability of zwitterionic PPDP gel electrolyte for solid-state supercapacitors under different pH. (a) Photographs of PPDP/H_2SO_4 (left) and PPDP/KOH (right) gel electrolytes at liquid state (top) and solid state (bottom). (b) CV curves of graphene-based solid-state supercapacitors applying PPDP/LiCl, PPDP/H_2SO_4 and PPDP/KOH gel electrolytes at liquid state and solid state, the scan rate is 100 mV s^{-1}.

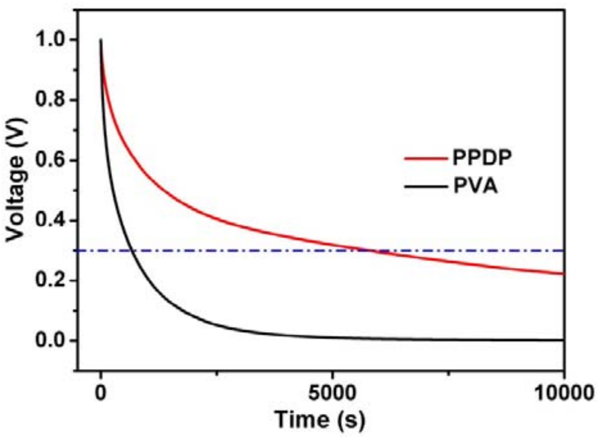

Supplementary Figure 12 | Self-discharging performance of the solid-state supercapacitors applying PPDP and PVA gel electrolytes

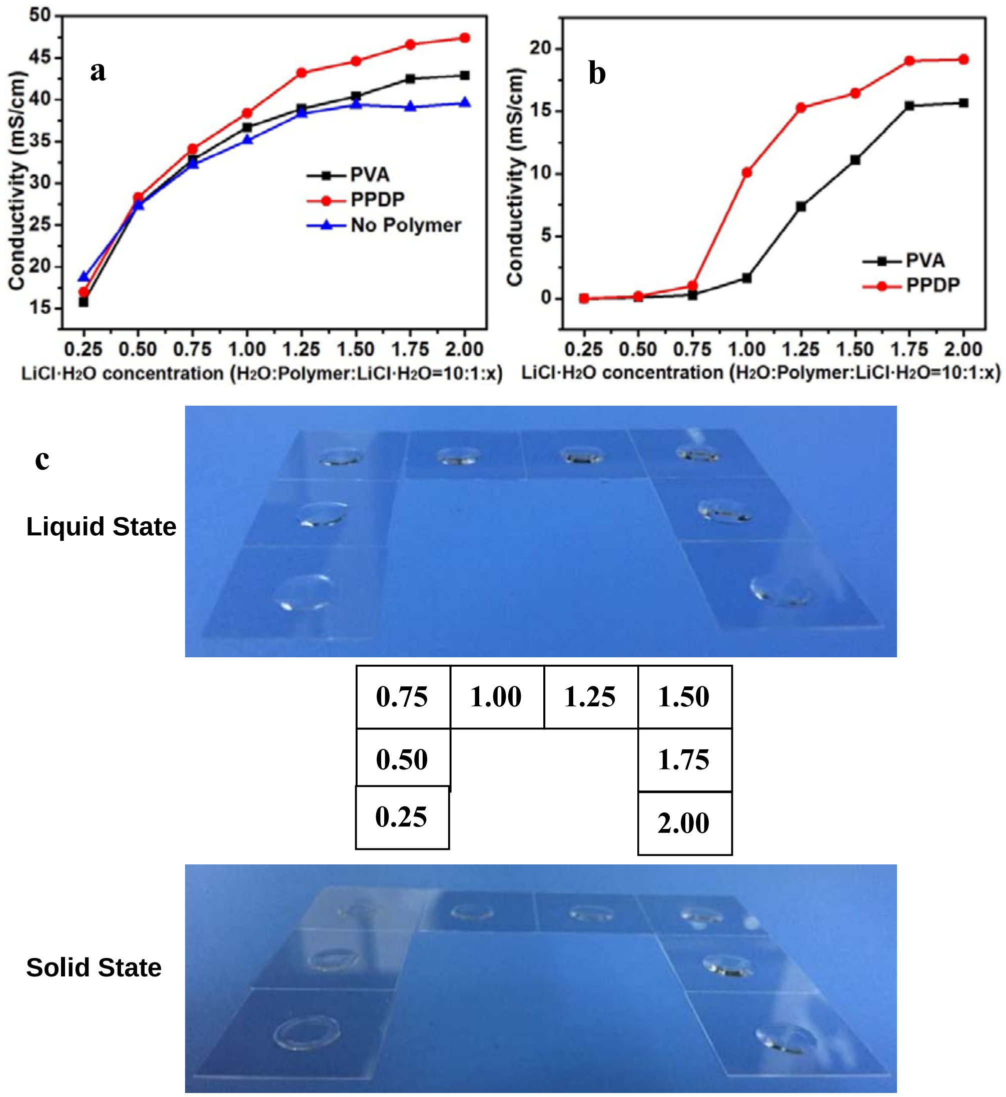

Supplementary Figure 13 | Ionic conductivity tests and physical states of the gel electrolytes with different salt concentration. (a) The ionic conductivities of PPDP and PVA gel electrolytes at liquid state with different salt concentration of LiCl•H_2O. “No polymer” represents the aqueous solution in the presence of different salt concentration of LiCl•H_2O without polymer. (b) The ionic conductivities of PPDP and PVA gel electrolytes at solid state with different salt concentration of LiCl•H_2O. (c) Photographs of PPDP gel electrolytes with different LiCl•H_2O concentration at liquid state (top) and after placing in ambient condition for 24 h (bottom).

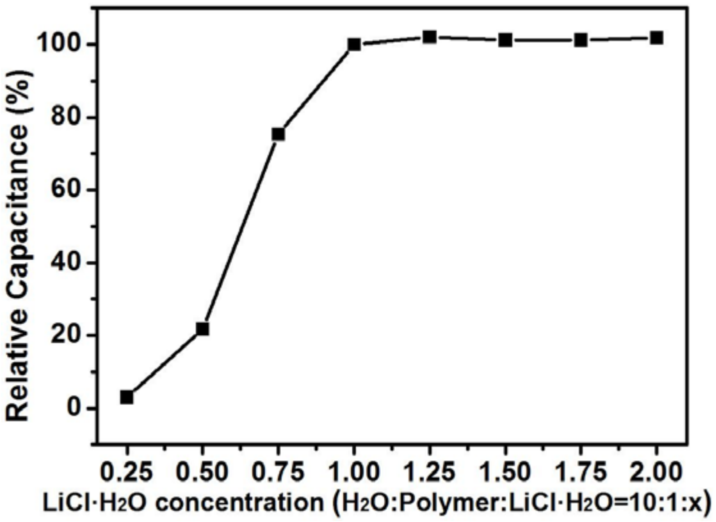

Supplementary Figure 14 | The influence of the concentration of LiCl•H_2O in PPDP gel electrolyte on the overall performance of supercapacitors. Relative specific capacitance of the solid-state supercapacitors with different concentration of LiCl•H_2O as the LiCl•H_2O/polymer ratio increases from 0.25 to 2.00.

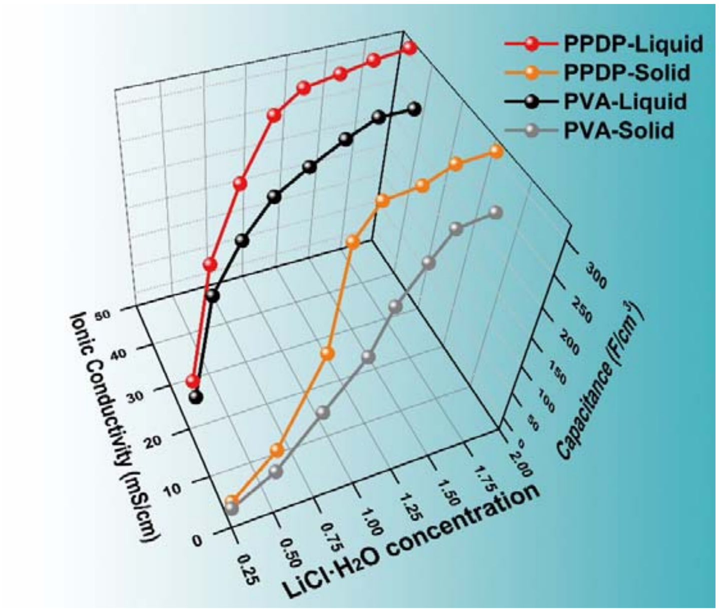

Supplementary Figure 15 | The ionic conductivity dependence of capacitance of the supercapacitors applying PPDP and PVA gel electrolytes at liquid and solid states as a function of the concentration of LiCl•H_2O.

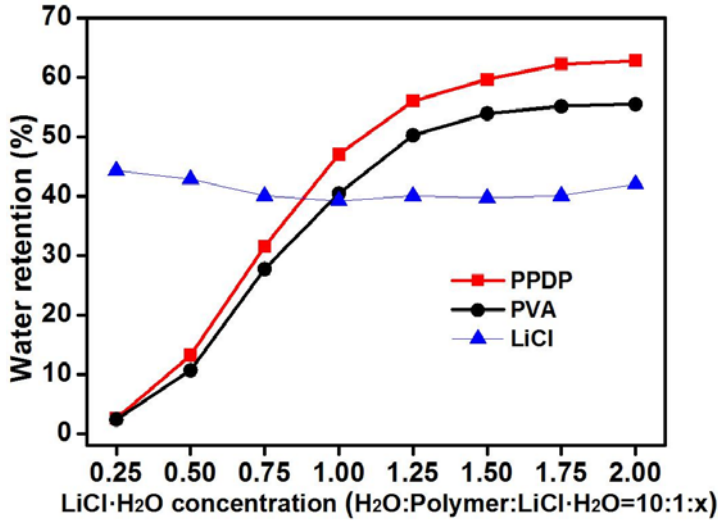

Supplementary Figure 16 | The role of LiCl in enhancing the water retention of gel electrolyte. Water retention test of the PPDP gel electrolyte, PVA gel electrolyte and LiCl aqueous solutions without polymer with different concentration of $LiCl \cdot H_2O$.

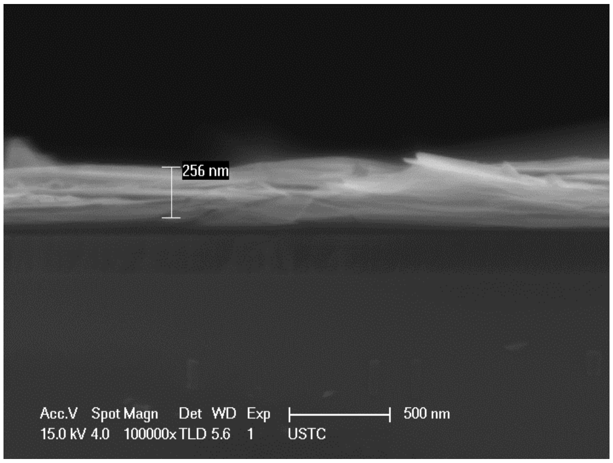

Supplementary Figure 17 | FE-SEM image of cross-sectional view of the as-fabricated graphene-electrodes. The cross-sectional FE-SEM image of graphene electrode shows that the graphene electrode used in our work is about 250 nm.

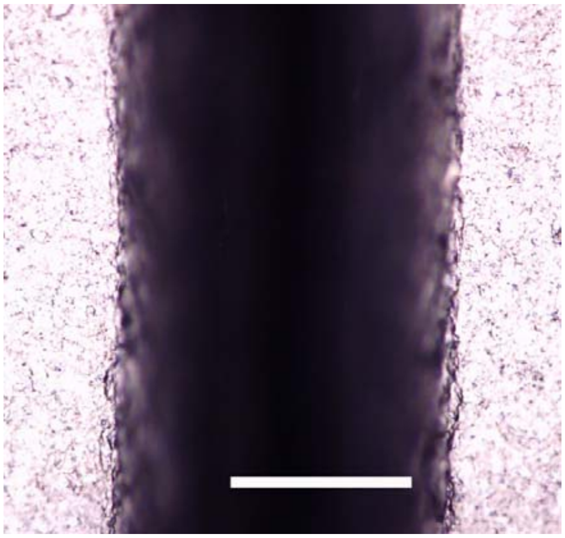

Supplementary Figure 18 | Optical image of the separation between the two electrodes. The distance between the two electrodes is ~150 μm. Scale bar: 100 μm.

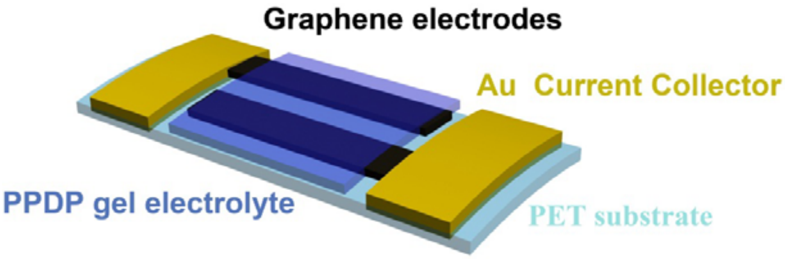

Supplementary Figure 19 | The architecture of solid-state supercapacitor applying PPDP gel electrolyte presented in this work.

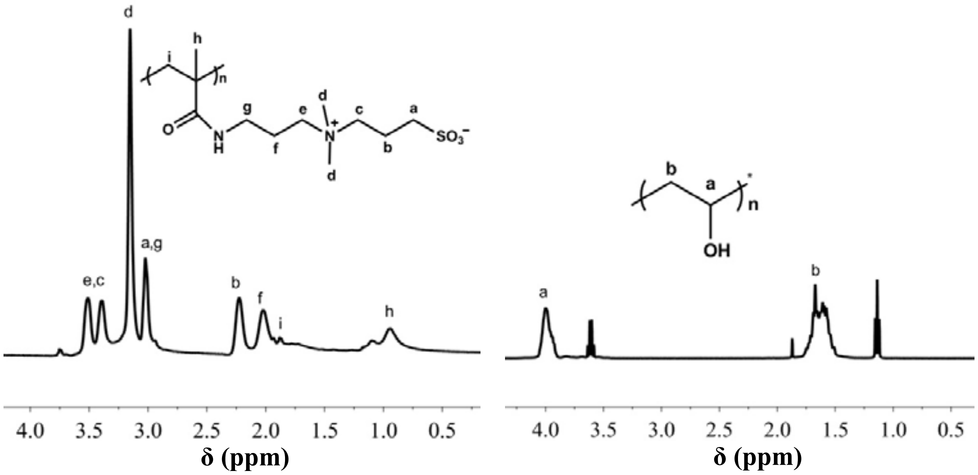

Supplementary Figure 20 | ^{1}H NMR spectra of PPDP and PVA.

Supplementary Table 1 | Viscosities of PPDP and PVA gel electrolytes at liquid state

Gel electrolytes	Viscosity η (Pa•s)
**PPDP/LiCl**	0.10
**PVA/LiCl**	0.91
**LiCl•H_2O**	1.39×10^{-3}

Supplementary Table 2 | The mass ratio of H_2O: polymer: $LiCl \cdot H_2O$ in gel electrolytes at liquid state and solid state

Gel electrolytes at liquid states	PPDP gel electrolytes at solid states	PVA gel electrolytes at solid states
**10:1:0.25**	0.03:1:0.25	0.03:1:0.25
**10:1:0.50**	0.23:1:0.50	0.18:1:0.50
**10:1:0.75**	0.81:1:0.75	0.67:1:0.75
**10:1:1.00**	1.78:1:1.00	1.36:1:1.00
**10:1:1.25**	2.86:1:1.25	2.27:1:1.25
**10:1:1.50**	3.70:1:1.50	2.93:1:1.50
**10:1:1.75**	4.53:1:1.75	3.38:1:1.75
**10:1:2.00**	5.07:1:2.00	3.75:1:2.00

Supplementary Note 1

It is known that cycling stability test was conducted by fast charging/discharging process with continuous ion migration in gel electrolyte. The zwitterionic side group would become more aligned undergo this process due to the external electric field (**Figure 1**). Therefore, the more well-aligned structure of zwitterionic side groups of PPDP gel electrolyte brought a better optimization of the ion migration channel, leading to a better electrochemical performance of the supercapacitors after numbers of cycling. In order to verify our opinion, we have conducted cyclic voltammetry test on solid-state supercapacitor applying PPDP gel electrolyte from 1st cycle to 10th cycle, as shown in **Supplementary Figure 4**. Apparently, the CV curves of solid-state supercapacitor applying PPDP gel electrolyte at 10 mV s^{-1} from the first 10 cycles shows the broadening of the CV response during the initial cycles, while the solid-state supercapacitor applying PVA gel electrolyte doesn't show this behavior. Considering that the structure and electrode materials are identical, the broadening of the CV response during initial process could be attributed to that the zwitterionic groups in the PPDP gel electrolyte become more aligned undergo the fast charging/discharging process, forming ion migration channel and thus the better electrochemical performance is achieved after numbers of cycling. As a result, the solid-state supercapacitor applying PPDP gel electrolyte has shown superior cycling stability than that of PVA gel electrolyte.

Supplementary Note 2

We have provided the viscoelastic properties of PVA gel electrolyte, as shown in **Supplementary Figure 6**. At the liquid state of PVA gel electrolyte, the storage modulus (G') is smaller than the loss modulus (G"), indicating the sol nature of the initial state of the PVA gel electrolyte. While the G' is larger than G" at the solid state after placing the gel electrolyte in ambient environment for 24 h, revealing that the PVA gel electrolyte transforms from liquid state into solid state.

Supplementary Note 3

The DSC thermograms shown in **Supplementary Figure 7** suggest that only 1-2 water molecules are tightly bound to one PVA monomeric unit *via* hydrogen bonding between the hydroxide group and the water molecules.

Supplementary Note 4

It is known that the strong hydration capacity of polyzwitterions is due to the electrostatic interactions (ion-dipole interactions) between the positively and negatively charged groups on the zwitterionic unit and the surrounding water molecules.[1-2] Hence, we have performed the low-field nuclear magnetic resonance (LF-NMR) measurements to further support the claim that the hydrated water molecules are tightly bound to the PPDP, through the electrostatic interactions according to the previous literature.[3] In **Supplementary Figure 8a**, the reciprocal of water relaxation time ($1/T_2$) decreases with increasing number of added water molecules per zwitterionic unit (N_w). When the surrounding water molecules are tightly bound to the zwitterionic group through the electrostatic interactions, the T_2 of bound water molecules becomes shorter compared with that of the bulk water molecules. Therefore, the number of bound water molecules can be obtained by utilizing the intersection of two straight lines drawn through the $1/T_2$ versus N_w curve. It can be seen that ~ 8 water molecules are strongly bound to one PDP unit via the electrostatic interactions as the T_2 in the range of $N_w < 8$ is shorter than that in the range of $N_w > 8$. Likewise, only ~ 1 water molecule is tightly bound to one PVA monomeric unit via the hydrogen bonding interactions (**Supplementary Figure 8b**). Thus, the LF-NMR results further confirm our claim that the PPDP gel electrolyte has higher water retention ability than the PVA gel electrolyte, which are well consistent with the DSC results presented in **Figure 4c**.

Supplementary Note 5

We have performed the water retention test of PPDP and PVA gel electrolytes under different salt concentrations to highlight the robust water retention ability of zwitterionic PPDP gel electrolyte. The water retention ability of PPDP gel electrolyte is superior than that of PVA gel electrolyte at the same salt concentration of $LiCl{\bullet}H_2O$. The water retention of PPDP gel electrolyte at solid state of 47.1 % is higher than that of the PVA gel electrolyte of 40.5 %. The value of water retention is calculated by dividing the mass of water remained within the gel electrolyte by the total mass of the gel electrolyte after placing in ambient condition for ~24 h until the equilibrium state was reached. As shown in **Supplementary Figure 9**, the water retention of the PPDP gel electrolyte has an obvious advantage over the PVA gel electrolyte at the same salt concentration ($LiCl{\bullet}H_2O$). When the ratio of $LiCl{\bullet}H_2O$ to polymer exceeds 1, the PPDP and PVA electrolytes cannot transform into solid-state due to the

excessive water content. Therefore, the inventory mass ratio of H_2O: polymer: LiCl•H_2O at 10:1:1 is chosen for preparing the gel electrolytes in this work.

Also, when placing gel electrolytes for a certain period of time in ambient condition, the water in gel electrolytes is partially evaporated. At last, the gel electrolytes transform into the solid state from the liquid state, and the water is no longer losing because of the high water retention ability of the polymers and the hygroscopic effect of the inorganic salt (LiCl•H_2O). Therefore, the final mass ratio of H_2O: polymer: LiCl•H_2O at solid state is no longer at 10:1:1, and we have provided the detailed ratios of PPDP and PVA gel electrolytes at solid state in **Supplementary Table 2**.

Supplementary Note 6

In order to further present the highly competent flexibility of PPDP gel electrolyte, we have conducted ionic conductivity test of PPDP gel electrolyte at solid state under different bending states as well as bending cycles, as shown in **Supplementary Figure 10**. The PPDP gel electrolyte shows good mechanical stability and flexibility during the bending tests, in which the conductivity of PPDP gel electrolyte remains almost constant under different bending angles (45º, 90º, 135º, 180º) (**Supplementary Figure 10a**). Meanwhile, the conductivity of PPDP gel electrolyte can also remain at about 10 mS cm^{-1} up to 1000 bending cycles, as shown in **Supplementary Figure 10b**. Therefore, the electrochemical stability of PPDP gel electrolyte at solid state was firmly verified by the superior mechanical stability and flexibility.

Supplementary Note 7

We have conducted the experimental data based on PPDP/H_2SO_4 and PPDP/KOH under different pH, as shown in **Supplementary Figure 11**, to demonstrate that PPDP has general applicability as gel electrolyte for solid-state supercapacitors under different pH. As shown in **Supplementary Figure 11a**, the pH is about 1 (left) for PPDP/H_2SO_4 gel electrolyte and is about 13 (right) for PPDP/KOH gel electrolyte at liquid state. Moreover, the liquid state gel electrolyte can transform into solid-state after placing in ambient condition for 24 h, forming physically crosslinked gel due to the dipole-dipole interactions between the zwitterionic groups, which is the same as the PPDP/LiCl gel electrolyte.

Furthermore, we have conducted PPDP/H_2SO_4 and PPDP/KOH gel electrolytes applying onto graphene-based electrodes to fabricate solid-state supercapacitors. The cyclic voltammetry (CV) has

been studied at the scan rate of 100 mV s^{-1}, as shown in Figure **Supplementary Figure 11b**. The performance of graphene-based solid-state supercapacitors applying PPDP/H_2SO_4 is comparable to that of the graphene-based solid-state supercapacitors applying PPDP/LiCl gel electrolyte. For the case of PPDP/KOH gel electrolyte, its performance of graphene-based solid-state supercapacitors is not as good as that of the PPDP/LiCl and PPDP/H_2SO_4 gel electrolytes. The capacitance retention ability of the PPDP/KOH gel electrolyte from liquid state to solid state is also lower than that of PPDP/LiCl and PPDP/H_2SO_4 gel electrolytes. The less excellent performance of the PPDP/KOH gel electrolyte compared to the other two kinds of gel electrolytes may be due to the influence of strong basic condition on the stability of amide part of PPDP, which would generate an impact on the ion migration channel of PPDP gel electrolyte, leading to a less excellent electrochemical performance in basic condition than that in the acidic and neutral conditions.

Supplementary Note 8

We have performed self-discharging measurement on the solid-state supercapacitors applying PPDP and PVA gel electrolytes. The self-discharging performance of solid-state supercapacitor applying PPDP gel electrolyte is much better than that of PVA gel electrolyte.

As shown in **Supplementary Figure 12**, the self-discharging process of solid-state supercapacitor applying PPDP gel electrolyte and PVA gel electrolyte is measured on galvanostatic charge-discharge curve from 1.0 V. After 5803 s the voltage of solid-state supercapacitor applying PPDP gel electrolyte decreases to 0.3 V, while the time required for the voltage of the solid-state supercapacitor applying PVA gel electrolyte decreases to 0.3 V is only 670 s. Considering that the structure and electrode materials are identical, the obvious difference in self-discharging process between PPDP and PVA gel electrolytes can be attributed to the superior ability of the PPDP gel electrolyte to retain charge and to retard the self-discharging process.

Supplementary Note 9

We have tested the ionic conductivity of the PPDP gel electrolyte at liquid state and solid state. The conductivity of gel electrolyte is 38.4 mS cm^{-1} at liquid state and 10.1 mS cm^{-1} at solid state, respectively.

Also, we have tested the ionic conductivity of zwitterionic gel electrolytes with different salt

concentration at liquid state and solid state, as shown in **Supplementary Figure 13a** and **Supplementary Figure 13b**. In our opinion, there is an optimized inventory (initially added) mass composition of H_2O: polymer: LiCl•H_2O of 10:1:1. We think that the salt concentration of LiCl•H_2O plays a key role in the ionic conductivity at liquid state and solid state. To verify our opinion, we have designed control experiments to study the effect of LiCl•H_2O concentration in the PPDP and PVA gel electrolytes on ionic conductivity under liquid state and solid state. As illustrated in **Supplementary Figure 13a**, the conductivity sharply increases with increasing mass ratio of LiCl•H_2O to polymer from 0.25 to 1. When the ratio exceeds 1.25, the ionic conductivities of PPDP and PVA gel electrolytes undergo negligible changes, and the conductivity of PPDP gel electrolyte is higher than that of PVA gel electrolyte at the same salt concentration. Meanwhile, as shown in **Supplementary Figure 13b**, after the gel electrolytes were placed in ambient condition for 24 h along with water evaporation, the gel electrolytes with the ratio less than 1 can transform into solid state, which is highlighted in the digital photograph of **Supplementary Figure 13c**. As the concentration of LiCl•H_2O increases in this range, the conductivity has an obvious increase before the ratio reaches 1 for the both two kinds of gel electrolytes, but the conductivity of PPDP gel electrolyte has an apparent advantage over that of PVA gel electrolyte. However, when the ratio exceeds 1, the water retention is higher than 50 %, which causes a serious problem that both the PPDP and PVA electrolytes cannot transform into solid-state. Therefore, we choose 10:1:1 as the best inventory mass ratio of H_2O: polymer: LiCl•H_2O in our work, which gives rise to a reasonable ionic conductivity as well as the good ability of the gel electrolytes to transform into solid-state for solid-state supercapacitors.

Supplementary Note 10

The concentration of inorganic salt in the PPDP gel electrolyte is one of the key factors to affect the overall performance of solid-state supercapacitors. We have conducted seven control experiments with different ratio of LiCl•H_2O to polymer equal to 0.25, 0.50, 0,75, 1.25, 1.50, 1.75, 2.00 to explore the influence of inorganic salt (LiCl•H_2O) in the PPDP gel electrolyte on the performance of graphene-based solid-state supercapacitors, as shown in **Supplementary Figure 14**. The specific capacitances acquired from the solid-state supercapacitors applying PPDP gel electrolytes with the ratio of 0.25, 0.50 and 0.75 display obvious disadvantages compared to that with the ratio of 1.00. Meanwhile, when the ratio of LiCl•H_2O to polymer in PPDP gel electrolytes increases from 1.25 to

2.00, the specific capacitances keeps almost constant. Considering that insufficient LiCl•H_2O concentration in gel electrolytes is unfavorable for the ionic conductivity and specific capacitance and the excess LiCl•H_2O concentration in gel electrolyte prevents the gel electrolytes from transforming into solid state due to the hygroscopic property, we have experimentally chosen the optimal inorganic salt concentration (LiCl•H_2O) in gel electrolyte with the inventory mass ratio of H_2O:PPDP:LiCl•H_2O of 10:1:1, at which the PPDP gel electrolyte has brought good performance in specific capacitance at solid state.

Supplementary Note 11

Indeed, there should be a certain relationship between ionic conductivity and capacitance. To clarify the relationship between ionic conductivity and capacitance, we have tested the influence of ionic conductivity of PPDP and PVA gel electrolytes at liquid and solid states on the capacitance of graphene-based solid-state supercapacitors mediated by the concentration of LiCl•H_2O, as shown in **Supplementary Figure 15**. Our results lead to the following two conclusions:

1. The capacitance is dominated by the ionic conductivity in the low ionic conductivity range of < 10 mS cm^{-1} (i.e. the concentration of LiCl•H_2O is less than 0.75). In this low salt concentration regime, the capacitance is mainly determined by the transportation and amount of the ions in the test system to realize double layer capacitance. In this case, the capacitance performance is restricted by ionic conductivity and salt concentration. Thus, the PPDP and PVA gel electrolytes at liquid state offer higher capacitances than those at solid state in the case of low salt concentration and low ionic conductivity.
2. Once the ionic conductivity is above 10 mS cm^{-1} (i.e. the concentration of LiCl•H_2O is higher than 1.0 in PPDP gel electrolyte), the capacitance performance is not determined by these factors any more, in that there are sufficient ions to realize double layer capacitance. In other words, the capacitance could be dominated by other factors including superior penetration behavior of PPDP and facile ion migration channel of PPDP gel electrolyte, which would bring higher capacitance for solid-state supercapacitors applying PPDP gel electrolytes than that for the liquid PVA-based system, even though the ionic conductivity of the former (10.1 mS cm^{-1}) is lower than that of the latter (36.0 mS cm^{-1}). This result can be further validated by the fact that the specific capacitances keep almost constant after the ionic conductivity and concentration of

$LiCl \cdot H_2O$ respectively exceeds 10 mS cm^{-1} and 1.0.

Supplementary Note 12

To explore the actual role of LiCl in water retention, we have conducted the water retention test with LiCl aqueous solution in the absence of polymer, as shown in **Supplementary Figure 16**. Seen from **Supplementary Figure 16**, we can get following experimental information about the role of LiCl in water retention:

1. **The water retention of gel electrolyte is not merely dependent upon the concentration of LiCl.**

Seen from **Supplementary Figure 16**, the LiCl aqueous solution without polymer has stable water retention of about 42 % ± 2 % with increasing LiCl concentration from 0.25 to 2.00 (0.42 to 3.33 mol L^{-1}), although the water retention of the PPDP gel electrolyte increased with the increase of LiCl. In this regard, the polymers also play an important role in achieving the high water retention of gel electrolytes, and the water retention of gel electrolytes is not merely dependent upon the concentration of LiCl.

2. **The polymers and LiCl play a synergic effect in achieving the enhanced water retention of gel electrolytes**.

The water retention of gel electrolytes is strongly affected by both polymers and LiCl through the competition of polymer-ion interactions with the polymer-water and ion-water interactions. In the low LiCl concentration regime, the polymer-ion interactions (e.g., ion-paring interaction and ion-dipole interaction) dominate over the polymer-water and ion-water interactions.[4-5] Therefore, both polymers and LiCl render gel electrolytes a low water retention ability. As the salt concentration increases, the gel electrolytes gradually exhibit good water retention ability due to the excess free Li^+ and Cl^-. As the PPDP gel electrolyte is physically crosslinked by the dipole-dipole interaction, the increase of LiCl concentration would increase the water retention ability of PPDP via weakening the dipole-dipole interaction. A similar mechanism can also be applied to the PVA gel electrolyte which is physically crosslinked by the hydrogen bonds. When the concentration of $LiCl \cdot H_2O$ is above 1, the PPDP electrolyte is over hydrated and cannot transform into solid-state due to the lack of sufficient dipole-dipole interactions.[3] In a word, systematical experiments have revealed that the polymers and LiCl play a synergic effect in achieving the enhanced water retention of gel electrolytes.

Supplementary Note 13

In our work, since the thickness of the electrodes is ~ 250 nm, as shown in **Supplementary Figure 17** and the surface area of the electrodes is ~ 10 mm×1 mm for each graphene electrode, the weight of the graphene electrodes is hard to precisely measure by a balance. Thus, the entire mass of the device mainly depends on the weight of PET substrate and PPDP gel electrolyte. In this regard, the entire mass of PET substrate and PPDP gel electrolyte in our device measured by a balance is ~ 0.3069 g. Hence, the as-fabricated supercapacitor delivers a gravimetric energy density of 681 mWh kg^{-1} at a gravimetric power density of 6.52 W kg^{-1}.

Supplementary Note 14

The ^{1}H NMR spectra of PPDP and PVA shown in **Supplementary Figure 20** demonstrate the chemical structures of the polymeric matrixes for the solid-state supercapacitors.[6-7]

Supplementary References.

1. Keefe, A.J & Jiang, S. Poly(zwitterionic)protein conjugates offer increased stability without sacrificing binding affinity or bioactivity. *Nat Chem*, **4**, 59-63 (2012).
2. Chen, S., Zheng, J., Li, L. & Jiang, S. Strong Resistance of Phosphorylcholine Self-Assembled Monolayers to Protein Adsorption: Insights into Nonfouling Properties of Zwitterionic Materials. *J. Am. Chem. Soc.* **127**, 14473-14478 (2005).
3. Wang, T., Wang, X., Long, Y. Liu, G. & Zhang, G. Ion-Specific Conformational Behavior of Polyzwitterionic Brushes: Exploiting It for Protein Adsorption/Desorption Control. *Langmuir*, **29**, 6588-6596 (2013).
4. Okur, H.I., Kherb, J. & Cremer, P.S. Cations Bind Only Weakly to Amides in Aqueous Solutions. *J. Am. Chem. Soc.* **135**, 5062-5067 (2013).
5. Salis, A. & Ninham, B.W. Models and mechanisms of Hofmeister effects in electrolyte solutions, and colloid and protein systems revisited. *Chem. Soc. Rev.* **43**, 7358-7377 (2014).
6. Geagea, R., Aubert, P.H., Banet, P. & Sanson, N. Signal enhancement of electrochemical biosensors via direct electrochemical oxidation of silver nanoparticle labels coated with zwitterionic polymers. *Chem. Commum.* **51**, 402-405 (2015).
7. Mori, Y., Yokoi, H. & Fujise, Y. Structural Investigation of Iron(III) and Copper(II) Complexes with Poly(vinyl alcohol) by NMR Techniques. *Polymer Journal* **27**(3), 271-279 (1995).
